# Derivation and Mapping of Critical Loads for Nitrogen and Trends in Their Exceedance in Germany

**DOI:** 10.1100/tsw.2001.330

**Published:** 2001-12-06

**Authors:** Hans-Dieter Nagel, Heinz-Detlef Gregor

**Affiliations:** Oeko-Data, Stausberg, Germany

**Keywords:** environmental management and policy, environmental modeling, environmental monitoring

## Abstract

The term “critical load” means a quantitative estimate of an exposure to one or more pollutants below which significant harmful effects on specified sensitive elements of the environment do not occur, according to present knowledge. In the case of nitrogen, both oxidised and reduced compounds contribute to the total deposition of acidity, which exceeds critical loads in many forest ecosystems. These also cause negative effects through eutrophication. Critical loads of nitrogen were derived for forest soils (deciduous and coniferous forest), natural grassland, acid fens, heathland, and mesotrophic peat bogs. In Germany, a decrease in sulphur emissions over the past 15 years resulted in a reduced exceedance of critical loads for acid deposition. In the same period it was noted that reduction in the emissions of nitrogen oxides and ammonia remained insignificant. Therefore, emissions of nitrogen compounds have become relatively more important and will continue to threaten ecosystem function and stability. The risk of environmental damage remains at an unacceptable level. The German maps show the degree to which the critical loads are exceeded, and they present current developments and an expected future trend. Results indicate that recovery from pollutant stress occurs only gradually.

## NITROGEN: INCREASING EVIDENCE OF AIR POLLUTION DAMAGE IN GERMANY

In Germany, in addition to the continuing concerns regarding human health, forest damage has been a well-documented case of environmental harm. Defoliation is still a widespread phenomenon in German forests and is monitored and assessed annually on a nationwide scale. Other fields requiring political action include soil acidification, eutrophication, and corrosion of material surfaces[1].

Nitrogen pollution has been a major concern in Germany because it increases risk to human health, causes forest damage, and produces a loss in biodiversity. Long-term deposition of air pollutants is one of the main factors in the processes linked to defoliation. More than a decade of well-organized research into the cause-and-effect relationships and their evaluation have succeeded in identifying the important contributors to these complex phenomena: (1) the deposition of acidifying and eutrophying pollutants and (2) episodes of elevated ambient ozone concentrations. Nitrogen, in particular, threatens the forests in Europe generally and in Germany specifically because of its (1) eutrophicating, potentially ecotoxic, acid-forming characteristic; and (2) its role as precursor to ozone[2].

Signed by 31 countries, the new United Nations Economic Commission for Europe (UN/ECE) Protocol to Abate Acidification, Eutrophication, and Ground-level Ozone sets reduction targets for four air pollutants including the most important acidifying and eutrophying components[3]. By 2010, Europe's sulphur emissions should be cut by 63%, $NO_x$ emissions by 41%, VOC

emissions by 40%, and ammonia emissions by 17% compared to their 1990 levels.

Each country's individual emission ceiling depends on (1) the impact that its emissions have on public health or ecological receptors and (2) the vulnerability of the environment that they pollute. The targets are set based on the simultaneous assessment of the effects of (1) $SO_2$ on acidity; (2) $NO_x$ on acidity, eutrophication, and human health via ozone formation; and (3) $NH_3$ on acidification and eutrophication. The German reduction plans reflect these considerations (Table 1).

In the decade between 1987 and 1997, sulphur emissions in Germany were drastically reduced. Today, the reduction targets called for by the Protocol are almost satisfied.

On the other hand, reduction in the emissions of nitrogen oxides and ammonia has been relatively small. The emissions of nitrogen compounds have become more important as sulphur emissions have decreased[4] (Fig. 1). Clearly, nitrogen emissions will continue to threaten ecosystem functions and stability.

## THE CRITICAL LOADS CONCEPT

For the protection of human health and the environment (and in the interest of precaution, abatement, and the mitigation of environmental impacts) environmental policy has to define specific targets and goals. These environmental goals are policy guidelines directed toward the achievement or protection of a specified environmental quality. Decisions regarding the quality to be achieved are subject to political value judgement. Therefore, in the process of defining targets for environmental policy, the following are of special importance: (1) research on the effects of pollution, (2) the development of cause-effect relationships, and (3) environmental monitoring. Integrating these items into the social, technical, and economic aspects of the overall public policy often requires painful compromises later in the process[5]. However, environmental goals can be pursued directly or stepwise in the longer term.

**TABLE 1**
**The Gothenburg Protocol Emission Reductions for Europe and Germany[3]**

| Pollutant | Emission cuts for Europe (percentage reduction to 1990) | Emission cuts for Germany (percentage reduction to 1990) |
|---|---|---|
| $SO_x$ | 63 % | 90 % |
| NOy | 41 % | 60 % |
| $NH_3$ | 17 % | 28 % |
| VOC | 40 % | 69 % |

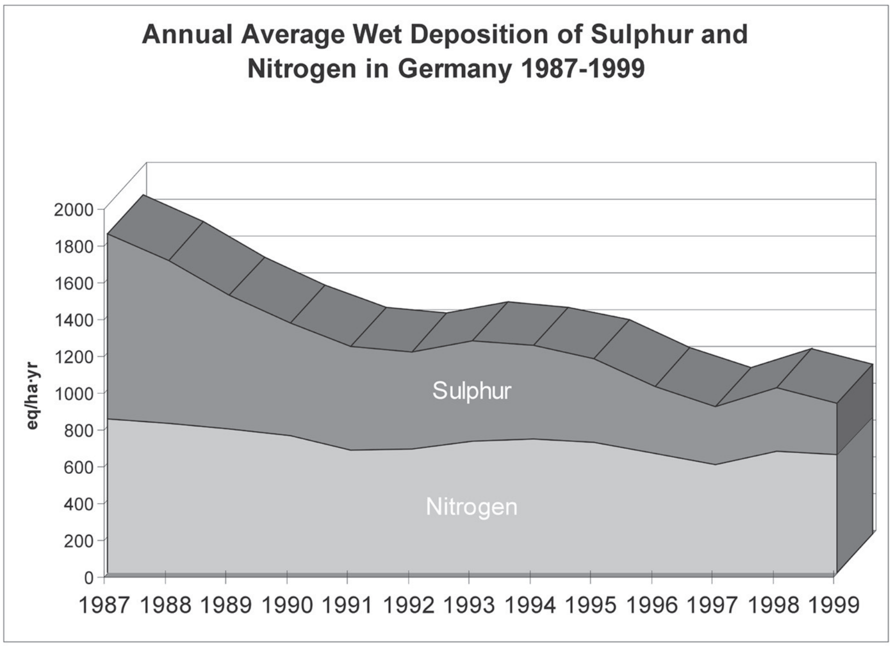

**FIGURE 1**. Wet deposition of acidifying compounds and the shares of sulphur and nitrogen.

Threshold values for the effects of air pollutants have attained much importance in the derivation of environmental goals. Their importance is based on the fact that they are criteria showing tolerable intensities of anthropogenic interference with ecosystems or the sensitive components of ecosystems. Critical loads and levels are such threshold values. By definition, these are measurable quantitative estimates showing the degree of tolerable exposure to one or more pollutants. According to present knowledge, when this exposure remains below the critical load threshold, significant harmful effects on specified environmentally sensitive elements can be avoided.

These values are purely scientific and unbiased but, while they reflect the state of present knowledge, are subject to revision as science develops further. The application of the critical loads concept is suitable to describe the limit to which a receptor system may be exposed to pollution without detectable damage in the long term or, alternatively, as the threshold below which present loading has to be reduced for recovery. For different receptors (in Germany the ecological receptors represent 30% of the total area; see Table 2), critical loads are computed and mapped. In Fig. 2, the critical load value is shown in the bottom of the column. The magnitude of the critical value depends on both the chosen receptor and the natural ecological conditions in the regarded area. Critical loads change over time only when changes in the receptor or in the predominating environmental conditions occur.

The actual load is the atmospheric deposition determined for a defined duration (e.g., a year). The actual load may be measured at the destination of the receptor itself or modelled based on surrounding data.

The critical load value is then compared to the actual load (deposition). The difference describes the exceedance, which has to be reduced to zero to protect the ecological receptor over time. In order to reach the zero exceedance threshold and protect the ecosystem, a stepwise reduction in exceedances is possible (target loads). These targets have become and will continue to be elements in national or international agreements. One common target is the gap closure between critical and actual loads by a percentage (e.g., 60%). Consequently, the source of pollution has to be identified and emission ceilings have to be set. The ultimate goal should be to reduce deposition to the critical loads.

The critical loads concept is a well-established element within the work of the UN/ECE Convention on Long-range Transboundary Air Pollution. The concept is, in fact, the scientific rationale for the development of effect-based air-pollution abatement strategies. As a concept, it helps to describe the limits to which ecosystems can cope with pollution. The concept is of special value to the aim of “sustainable development”, an aim that was first introduced by Hartig in 1795 into German forestry[6] but now guides environmental policy in general.

**TABLE 2**
**Ecological Receptors for the Critical Load Approach in Germany**

| Ecosystem types | Percentage of receptor area to the total area of Germany [%] | Percentage of the total receptor area [%] |
|---|---|---|
| deciduous forest | 6,5 | 21,6 |
| coniferous forest | 16,0 | 52,9 |
| mixed forest | 6,4 | 21,3 |
| natural grassland | 0,5 | 1,8 |
| acid fens and heathland | 0,3 | 1,0 |
| wet grassland | 0,1 | 0,4 |
| mesotrophic peat bogs | 0,3 | 1,0 |
| | 30,1 | 100 |

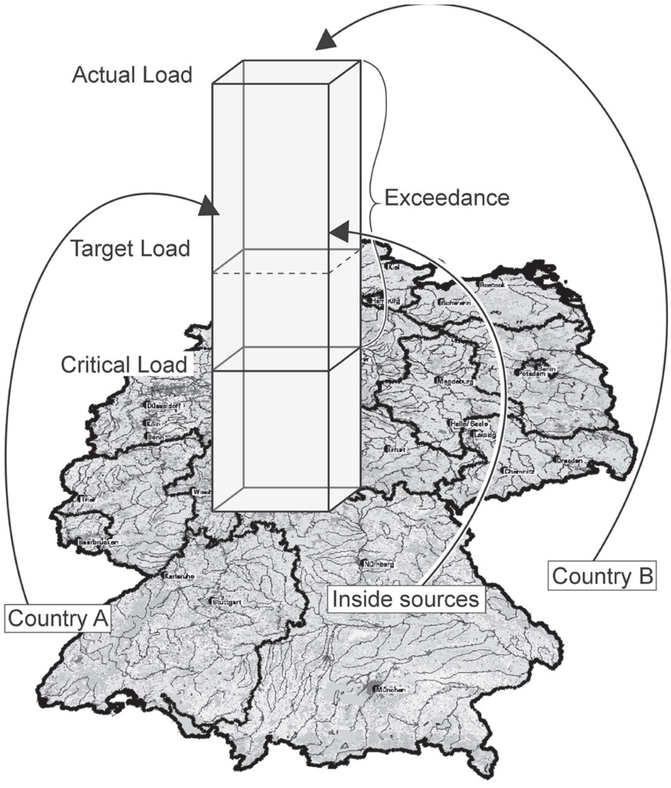

**FIGURE 2.** The critical load concept.

## CRITICAL LOADS FOR NITROGEN

Nitrogen affects ecosystems in different ways: it causes acidification and is responsible for eutrophication and the formation of ground-level ozone. According to the general definition of the critical loads (as ecologically based environmental objectives for airborne pollutants), the thresholds of an input of acidity [$CL_{max}$(S), $CL_{max}$(N)] must be calculated as well as the input of nutrient nitrogen [$CL_{nut}$(N)] which will not cause any long-term adverse effect to structure and/or function of the ecosystems. The magnitude of the critical load value should depend only on the characteristics of the regarded ecosystems. Changes due to acid deposition, which are responsible for damage to structure and function of forest and other natural ecosystems, can be identified using parameters of the chemical composition of the soil solution. Significant damage can be expected to develop when certain chemical parameters of the soil solution show a marked deviation from the steady-state conditions. Such deviations lead to destabilization of soil processes or to direct vegetation damage.

Eutrophication will occur when nitrogen inputs result in nutrient imbalances. When the natural nutrient supply becomes unbalanced, the ecosystem is affected by climatic and other stresses and may loose its natural biodiversity.

The critical loads methodology is comprehensively described in the "Mapping Manual"[7] of the UN/ECE International Co-operative Programme on Mapping Critical Loads and Levels (ICP Mapping). The manual is accessible via the Internet at URL: http://www.icpmapping.com.

Through following this manual, all necessary input parameters and indicators can be measured or derived in a way that enables national mapping exercises at different levels of sophistication. The German Mapping Programme is carried out by the National Focal Centre of the ICP Mapping at the Federal Environmental Agency (UBA) in cooperation with Stuttgart University and the OEKO-DATA Company.

For the determination of critical loads in Germany, as in most of the other European countries, a simple mass balance is used. To derive the acidifying effects of nitrogen in this mass balance, the acid neutralizing processes within the ecosystem are compared with the anthropogenic acid deposition. The following indicators (derived from observed effects) were found suitable in the German national critical loads mapping programme to determine a critical status of acidification: (1) aluminium concentration, (2) base-cations/aluminium ratio, (3) pH value, (4) base saturation of soils, and (5) acid neutralizing capacity. The mass balance equations for the maximum critical load for sulphur-based acidity, $CL_{max}$(S), and the maximum critical load for nitrogen-based acidity, $CL_{max}$(N), are given in Fig. 3. For acidity problems in general, the map of critical loads in terms of sulphur, $CL_{max}$(S), is always used.

The release of base cations by natural weathering is assumed to be the most relevant process for buffering acid inputs to a forest ecosystem. Therefore the weathering process corresponds to the acid neutralizing capacity of the soil matrix. It can be quantified (as an estimate) on the basis of data on the parent material and the soil texture. The annual weathering rates are calculated individually for every soil horizon, and a weighted mean is

$$CL_{\max}(S) = BC^*_{dep} - Cl^*_{dep} + BC_w - BC_u - ANC_{le_{(crit)}}$$

$$CL_{\max}(N) = \frac{N_u + N_i + CL_{\max}(S)}{(1 - f_{de})}$$

| | |
|---|---|
| ***$CL_{max}$(S)*** | ***The critical load for sulphur-based acidity*** |
| ***$CL_{max}$(N)*** | ***The critical load for nitrogen-based acidity*** |
| ***$BC_w$*** | ***Base cations weathering derived from soil type and parent material class*** |
| ***$BC^*_{dep}$*** | ***Base cations deposition (sea salt corrected)*** |
| ***$Cl^*_{dep}$*** | ***Deposition of chloride (sea salt corrected)*** |
| ***$BC_u$*** | ***Base cations uptake and removal by biomass under steady state conditions*** |
| ***$N_u$*** | ***Nitrogen uptake and removal by biomass under steady state conditions*** |
| ***$N_i$*** | ***Long-term immobilisation of nitrogen*** |
| ***$f_{de}$*** | ***Denitrification factor derived from the soil type, (between 0 and 1, lower and higher denitrification)*** |
| ***$ANC_{le\ (crit)}$*** | ***Acceptable leaching of acid neutralisation capacity*** |

**FIGURE 3**. Derivation of critical loads for acidity (sulphur and nitrogen).

derived from these values to describe the weathering rate of the upper 0.5 m of the soil. Other processes that are considered in the mass balance are the deposition of base cations (sea salt corrected), the net uptake of base cations by the vegetation, and the leaching of acid neutralizing capacity.

In Germany, the determination of critical loads for ecosystems initially centred on forests. Today, however, other natural ecosystems are involved. These include: (1) natural grassland, (2) acid fens and heathland, (3) wet grassland, and (4) mesotrophic peat bogs (Table 2).

The mass balance equation is also used to calculate critical loads for nutrient nitrogen (Fig. 4) and sums up all sources and sink processes of nutrient nitrogen in the considered ecological system. In this case, the nitrogen-fixing processes (immobilization, nitrogen uptake in the biomass) and nitrogen removal (denitrification, acceptable nitrogen leaching) should be in balance with the nitrogen deposition for steady-state conditions.

Since sulphur and nitrogen behave differently within ecosystems, it is impossible to define a unique critical load for acidity and eutrophication. However, the combination of sulphur and nitrogen depositions not causing harmful effects lie within the critical load function[8] as shown in Fig. 5. For a given deposition of sulphur and nitrogen compounds, an exceedance may be defined as the sum of deposition reductions required to achieve nonexceedance by taking the shortest path to the critical load function. In practice, external factors such as the costs of emission reductions also determine which path will be followed to reach zero exceedance.

The function is computed for all ecological receptors. The typical view of one dataset, presented here as a generalised example indicates the downward movement of the deposition point towards the sulphur threshold; at present being larger than the gap between the nutrient critical load and the nitrogen deposition becoming smaller (Fig. 5).

$$CL_{nut}(N) = N_u + N_i + N_{le_{(acc)}} + N_{de}$$

| | |
|---|---|
| $CL_{nut}(N)$ | ***The critical load for nutrient nitrogen*** |
| $N_u$ | ***Nitrogen uptake and removal by biomass under steady state conditions*** |
| $N_i$ | ***Long-term immobilisation of nitrogen*** |
| $N_{le(acc)}$ | ***Acceptable leaching of nitrogen*** |
| $N_{de}$ | ***Denitrification*** |

**FIGURE 4**. Derivation of critical loads for eutrophication (nutrient nitrogen).

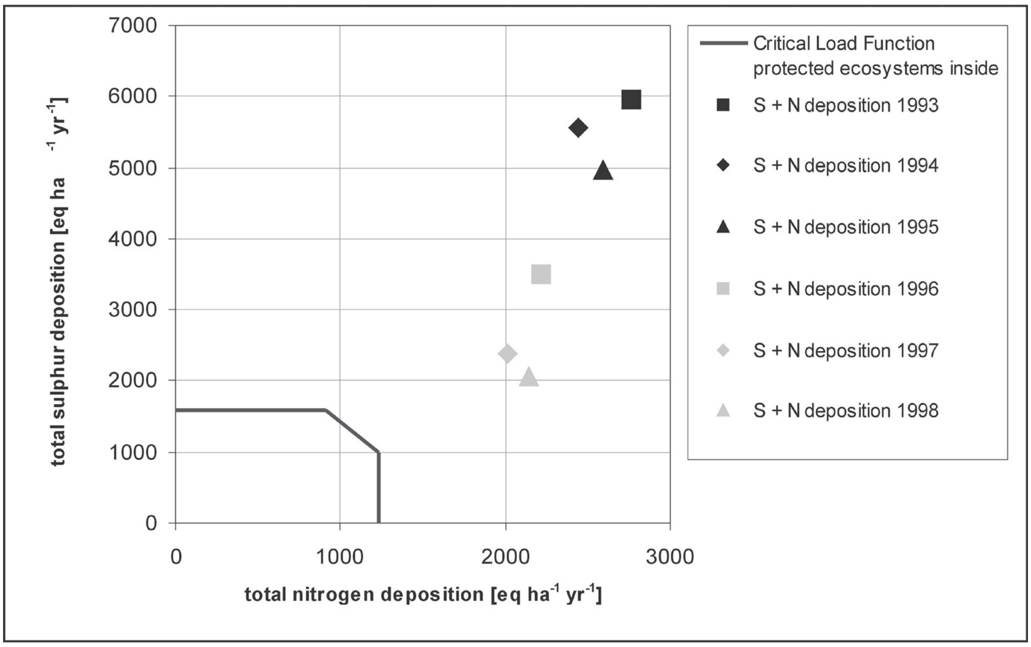

**FIGURE 5**. Critical load function for a single German dataset (generalised).

The German critical load database consists of 424.026 records; the regional distribution is mapped for acidity (Fig. 6) and nutrient nitrogen (Fig. 7).

## TRENDS OF ACTUAL DEPOSITION AND CRITICAL LOAD EXCEEDANCES

Since the UN/ECE protocols on reducing emissions of sulphur (signed in 1985) and nitrogen (1988) have come into force, ever-increasing resources have been directed to develop effect-based approaches to control air pollution. With the implementation of the second Sulphur Protocol (1994) and, more recently, the Gothenburg Protocol (1999)[3], critical loads have been applied as guidelines for abatement strategies in Europe and in Germany. During the last decade, significant progress was made in calculating critical loads for nitrogen. These values have been compared to actual deposition load values. Due to the transboundary transportation of air pollution, an international approach is necessary to solve the problems and achieve recovery in ecosystems. Therefore, within the framework of the Convention,

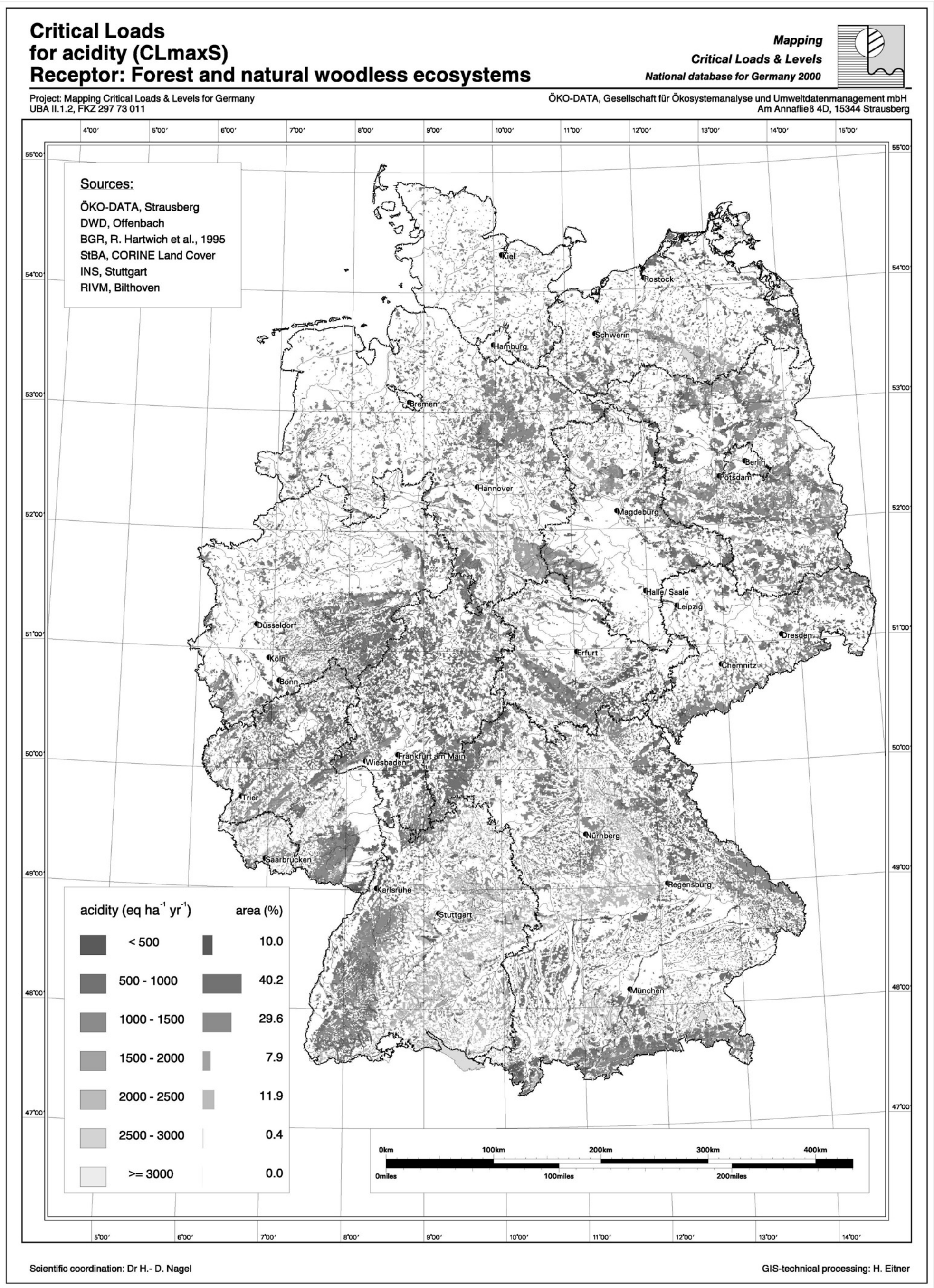

**FIGURE 6**. Regional distribution of critical loads for acidity.

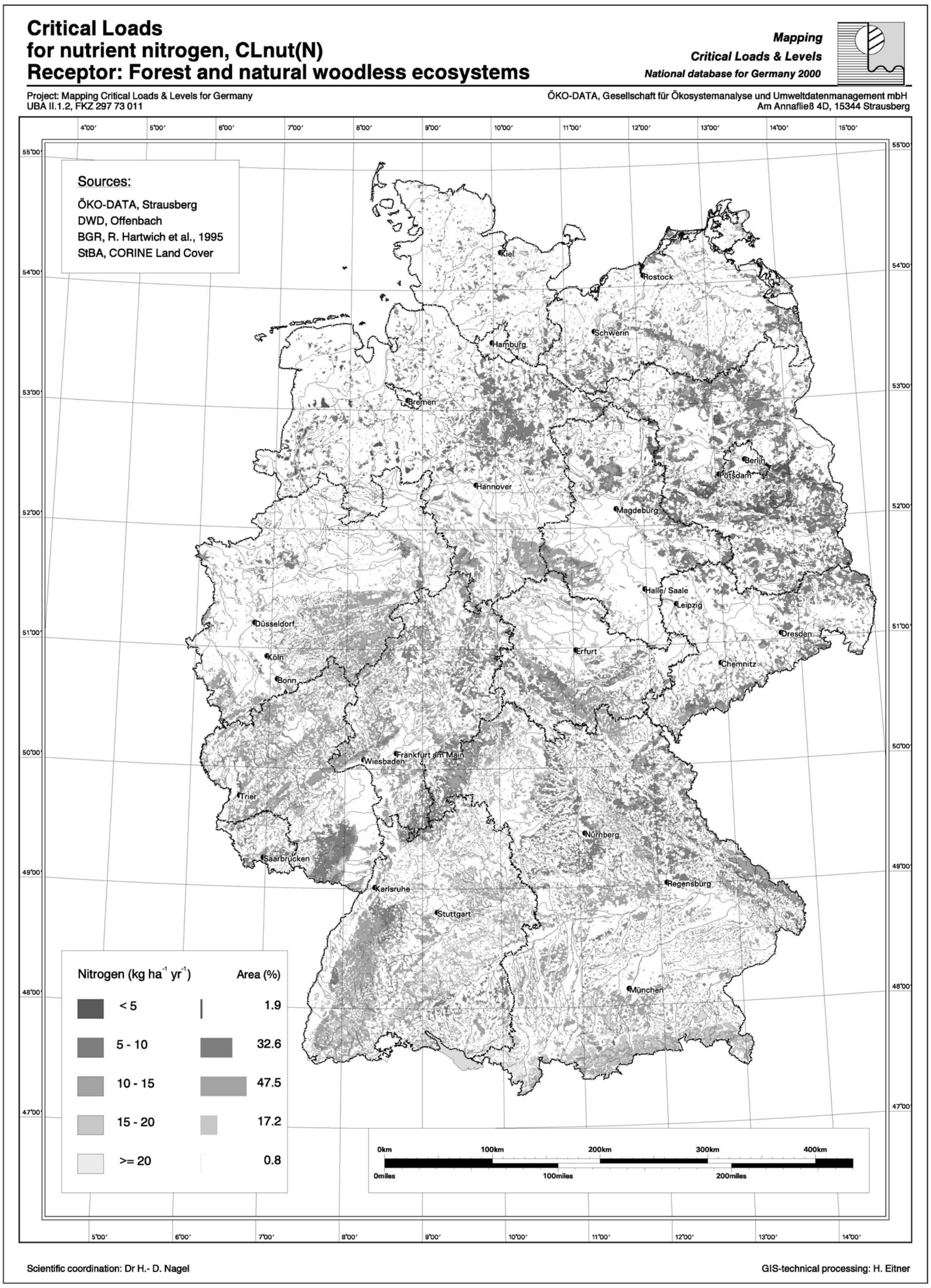

**FIGURE 7**. Regional distribution of critical loads for nutrient nitrogen.

Germany has established intensive cooperation with the neighbouring countries to develop harmonized methods using the critical loads concept.

In the middle of the last decade, countries in Europe observed the first results of the emission reduction promoted by the UN/ECE protocol activities. In all Europe, the area of unprotected receptors fell from 30% in 1980 to 10% in 1995. At the same time, critical loads for acidity in Germany were still markedly exceeded. The area of unprotected ecosystems still amounts to almost 99%. However, recently enacted measures are becoming effective. Additional commitments have brought about proof that the degree of acid's critical load exceedance in Germany is falling. It is predicted to fall further in the future. The share of receptor areas with extremely high exceedances of 5000 Eq $ha^{-1}$ $year^{-1}$ or more has decreased from about 80% (1987) to below 1% (1998), and the full implementation of the Gothenburg Protocol in the year 2010 will lead to further improvements. It is expected that about 70% of the receptor area will be close to critical loads in the near future (Fig. 8).

In the past decade, the exceedance of critical loads for nutrient nitrogen was continuously high and stressed all ecological receptors under consideration. Fig. 9 makes the extent of the exceedance visible. Regions experiencing the highest exceedances of critical loads for nitrogen have decreased. However, there is

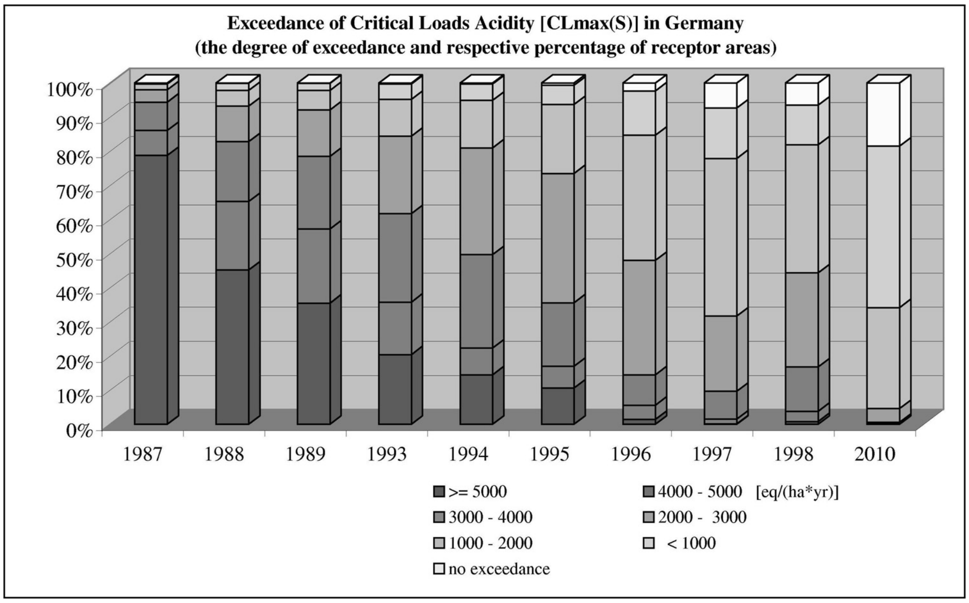

**FIGURE 8**. Exceedance of critical loads for acidity determined for 1987–1998 and estimated for 2010, after the full implementation of the Gothenburg Protocol.

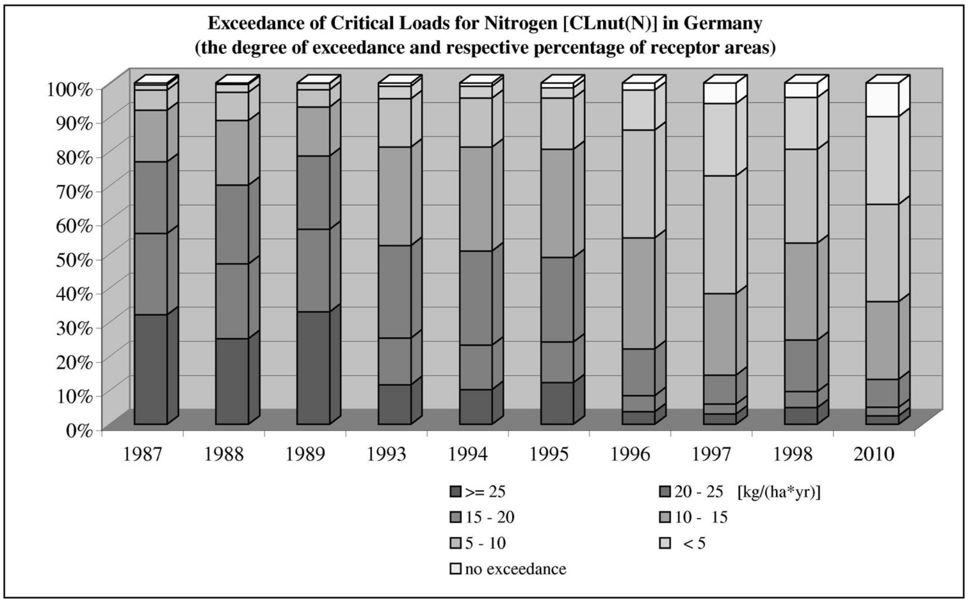

**FIGURE 9**. Exceedance of critical loads for nutrient nitrogen determined for 1987–1998 and estimated for 2010, after the full implementation of the Gothenburg Protocol.

no overall downward trend indicating a decrease in the total area affected by excess nitrogen deposition in Germany.

## CONCLUSIONS

In Germany, although a substantial reduction in sulphur emissions has been achieved and a corresponding decrease in acid deposition, nitrogen deposition still remains the main contributor to the unacceptable level of acidification and eutrophication. Due to the efforts of environmental protection during the last years, the trends in the exceedance of critical loads, both for acidity and eutrophication, are reflecting a reduced stress by air pollution. However, there has been no recovery. The percentage of ecological receptors with the highest exceedance is becoming increasingly smaller. From the peak in 1985, when critical loads in the whole area of mapped ecosystems were exceeded and about 80% of them exhibited extremely high values, the exceedance of critical loads has fallen. This decrease in the extent of exceedance is mainly due to a substantial reduction in sulphur emissions.

However, the emission of nitrogen oxides and ammonia has not decreased sufficiently and thus will continue to contribute to acidification and eutrophication. This fact, along with the acidity already accumulated in the soils, will remain responsible for the continued environmental problems in forest soils and other natural ecosystems in the coming decade. Studies based on the derivation and mapping of critical loads for acidity and eutrophication show that recovery from pollutant stress will often be very slow and may sometimes even take a hundred years.

| | | |
|---|---|---|
| Received: | July | 20, 2001 |
| Revised: | October | 18, 2001 |
| Accepted: | October | 29, 2001 |
| Published: | December | 6, 2001 |
